# Acute Myocardial Infarction and Pulmonary Diseases Result in Two Different Degradation Profiles of Elastin as Quantified by Two Novel ELISAs

**DOI:** 10.1371/journal.pone.0060936

**Published:** 2013-06-21

**Authors:** Helene Skjøt-Arkil, Rikke E. Clausen, Lars M. Rasmussen, Wanchun Wang, Yaguo Wang, Qinlong Zheng, Hans Mickley, Lotte Saaby, Axel C. P. Diederichsen, Jess Lambrechtsen, Fernando J. Martinez, Cory M. Hogaboam, MeiLan Han, Martin R. Larsen, Arkadiusz Nawrocki, Ben Vainer, Dorrit Krustrup, Marina Bjørling-Poulsen, Morten A. Karsdal, Diana J. Leeming

**Affiliations:** 1 Nordic Bioscience A/S, Herlev, Denmark; 2 School of Endocrinology, University of Southern Denmark, Odense, Denmark; 3 Institute of Clinical Research, Odense University Hospital, Odense, Denmark; 4 Nordic Bioscience Beijing, Beijing, China; 5 Department of Cardiology, Odense University Hospital, Odense, Denmark; 6 Department of Cardiology, Svenborg Hospital, Svenborg, Denmark; 7 Division of Pulmonary and Critical Care Medicine and Department of Pathology, University of Michigan, Ann Arbor, Michigan, United States of America; 8 Department of Biochemistry and Molecular Biology, University of Southern Denmark, Odense, Denmark; 9 Department of Pathology, Rigshopitalet, University of Copenhagen, Copenhagen, Denmark; University of Giessen Lung Center, Germany

## Abstract

**Background:**

Elastin is a signature protein of the arteries and lungs, thus it was hypothesized that elastin is subject to enzymatic degradation during cardiovascular and pulmonary diseases. The aim was to investigate if different fragments of the same protein entail different information associated to two different diseases and if these fragments have the potential of being diagnostic biomarkers.

**Methods:**

Monoclonal antibodies were raised against an identified fragment (the ELM-2 neoepitope) generated at the amino acid position ‘552 in elastin by matrix metalloproteinase (MMP) −9/−12. A newly identified ELM neoepitope was generated by the same proteases but at amino acid position ‘441. The distribution of ELM-2 and ELM, in human arterial plaques and fibrotic lung tissues were investigated by immunohistochemistry. A competitive ELISA for ELM-2 was developed. The clinical relevance of the ELM and ELM-2 ELISAs was evaluated in patients with acute myocardial infarction (AMI), no AMI, high coronary calcium, or low coronary calcium. The serological release of ELM-2 in patients with chronic obstructive pulmonary disease (COPD) or idiopathic pulmonary fibrosis (IPF) was compared to controls.

**Results:**

ELM and ELM-2 neoepitopes were both localized in diseased carotid arteries and fibrotic lungs. In the cardiovascular cohort, ELM-2 levels were 66% higher in serum from AMI patients compared to patients with no AMI (p<0.01). Levels of ELM were not significantly increased in these patients and no correlation was observed between ELM-2 and ELM. ELM-2 was not elevated in the COPD and IPF patients and was not correlated to ELM. ELM was shown to be correlated with smoking habits (p<0.01).

**Conclusions:**

The ELM-2 neoepitope was related to AMI whereas the ELM neoepitope was related to pulmonary diseases. These results indicate that elastin neoepitopes generated by the same proteases but at different amino acid sites provide different tissue-related information depending on the disease in question.

## Introduction

Elastin is an integral component of the extracellular matrix and enables tissues to repeatedly distend and relax over a lifetime. Elastin is predominantly expressed in connective and vascular tissues, in the lungs and in skin. Elastin makes up the core of elastic fibres and is surrounded by a mantle of fibrillin-rich microfibrils [1]. Loss of elasticity is a pathological feature of a number of degenerative and inflammatory diseases including vascular aneurysms [2], [3] and chronic obstructive pulmonary disease (COPD) with co-existing emphysema [4], [5]. For instance, deletion of the elastin gene in mice revealed lungs with emphysema-like lesions [6]. The primary tropoelastin transcript undergoes tissue-specific alternative splicing, which allow small adjustments of the functional properties of elastic fibres in different tissues [7]. In the arteries, elastin is responsible for vessel resistance to blood pressure. It is present in the medial layer, forming concentric fenestrated lamellae giving elasticity and resilience to the vessel walls [1]. In the lung, elastin is present in bronchi and lung parenchyma. In the alveolar regions of the lung, elastin fibers are deployed around alveolar ducts, the openings of alveoli and extensions into alveolar septa [8].

Extracellular matrix (ECM) components are degraded by a number of different proteases including matrix metalloproteinases (MMPs). Human neutrophil elastase (HNE), MMP-9 and MMP-12 in particular have been associated with elastin degradation and hence with cardiovascular [9]–[12] and respiratory diseases [13]–[15]. MMP-9 is over-expressed in alveolar macrophages in patients with idiopathic pulmonary fibrosis (IPF) [16]. MMP-9 has also been shown to be elevated in patients with acute coronary syndrome [17], [18] and MMP-9 and −12 have been pharmacogenetically associated with hypertension [19].

Macrophages are one of the main sources of MMPs in adult tissue. MMP-12 knock-out mice subjected to cigarette smoking do not have increased numbers of macrophages in their lungs and do not develop emphysema [20]. It has also been shown that MMP-12 production by macrophages plays a role in the transition of fatty acids to fibrous plaques during the progression of atherosclerosis [21] and that macrophages are implicated in all stages of myocardial infarction including inflammation, scar formation and tissue remodelling [22].

The cross-linked nature and extreme hydrophobicity of elastin makes it one of the most stable proteins in the body with a half-life of decades and subject to very little remodelling in healthy tissue. [23], [24] However, during inflammation the active macrophage-derived MMPs damage the microfibrils and the elastin core resulting in ravelled, disorganised elastin fibres [25]–[27] and degradation fragments. The degradation fragments are released into the circulation and can be measured in body fluids providing potential non-invasive biomarkers. These neoepitopes represent a unique fingerprint of proteolytic cleavage of the protein and may be used to identify whether the tissue is pathologically affected [28], [29]. Neoepitopes have been proven to be more accurate predictors of disease than their unmodified intact protein origin [30]–[34]. Measurement of different sub-pools of fragments from the same protein has yielded different information, emphasizing the importance of distinguishing each sub-pool from others [29], [33], [35]–[37].

Atherosclerosis is the underlying basis of most cardiovascular diseases (CVD) and degradation of extracellular proteins is a critical step for the development of atherosclerosis [38]. Asymptomatic remodeling of the arteries is initiated somewhat prior to clinical onset [39], making atherosclerosis difficult to diagnose. One biomarker or a panel of biomarkers may prove valuable in a clinical setting to detect early-stage atherosclerosis and trigger early intervention to potentially prevent complete blockage of the arteries [40]. Troponin has been found to be a cardiac-specific marker, but it is only useful in detecting myocardial damage in the late stages [41], [42]. Most other known CVD markers are related to specific risks, such as increased levels of lipids, the presence of diabetes, or inflammation, which can be detected by C-reactive protein. The widely used HeartScore for risk estimation combines smoking, age, gender, blood pressure and lipids [43]. Another score, the Agatstonscore measure calcified areas in coronary arteries measured by multidetector computed tomography [44]. Very few markers have so far been based on the composition of the atherosclerotic plaque.

The hypothesis for this research was that elastin is subject to pathologic remodeling during cardiovascular and pulmonary diseases, thus the aim was to investigate if two different fragments of the same protein entail different information associated to two different diseases and if these fragments have the potential of being diagnostic biomarkers. In the current study, a human elastin neoepitope, ELM-2, located at glycine ‘552 was identified and characterized. Its clinical relevance when assessed in serum was evaluated by ELISA in a cardiovascular study and in patients with COPD or IPF. Another elastin neoepitope, ELM, located at alanine ‘441, has recently been shown by our group to be elevated in patients with COPD or IPF [45]. ELM-2 was compared with ELM in both cardiovascular and pulmonary disease cohorts to investigate whether these two sub-pools of elastin fragments provide different information.

## Methods

### *In vitro* cleavage of purified elastin from human tissue

Purified elastin from human aorta (Sigma Aldrich), prepared using the method described by Starcher et al [46] was cleaved with MMP-1, MMP-9, cathepsin K, cathepsin S (Calbiochem, VWR), MMP-3, MMP-8, MMP-12 (Abcam), ADAMTS-1, −4 and −8 (Abnova).

The proteases were activated according to the manufacturers' instructions. Each cleavage was performed in a solution of protein/proteinase at a ratio of 100∶1. The activated proteinase was added to a MMP-buffer (100 mM Tris-HCl, 100 mM NaCl, 10 mM CaCl_2_, 2 mM ZnOAc, pH 8.0)), a cathepsin buffer (50 mM NaOAc, 20 mM L-cystine, pH = 5.5) or a aggrecanase buffer (50 mM tris-HCl, 10 mM NaCl, 10 mM CaCl_2_, pH = 7.5). The final concentration of elastin before cleavage was 0.33 mg/mL. Each aliquot was incubated for 2, 4, 24 and 48 hours at 37°C. All MMP cleavages were terminated using GM6001 (Sigma-Aldrich) and all cathepsin and aggrecanase cleavages using E64 (Sigma-Aldrich). Finally the cleavage was visually verified by using the SilverXpress® Silver Staining Kit (Invitrogen) according to the manufacturers' instructions.

Using the same procedure as described above, purified elastin from lung and aorta (Sigma Aldrich), prepared using the method described by Starcher et al [46], and solubilized elastin from aorta and lung (Sigma Aldrich), prepared using the method described by Partridge et al [47] were cleaved with MMP-9 and −12.

Human vascular tissue (atheroma-aorta, Biocat, Heidelberg, Germany) was cleaved by MMP-9 as described by Zhen et al [48]. Digestion was carried out at 37°C by adding 1 µg activated MMP-9 in 250 µL digestion buffer (1 M Tris buffer (pH 7.4), NaCl, CaCl_2_, ZnOAc). Supernatants were removed on days 1, 3, 7 and 10 and frozen at −80°C. At each time point, MMP-9 in digestion buffer was added to the vascular wall sample after removing the supernatants and incubation was continued.

### Peptide identification by mass spectrometry

The peptides were purified and desalted using reversed phase (RP) micro-columns (Applied Biosystems) prior to nanoLC-MS-MS analysis as described in the literature [49]. The purified peptides were re-suspended in 100% formic acid, diluted with H_2_O and loaded directly onto an 18 cm RP capillary column using a nano-Easy-LC system (Proxeon, Thermo Scientific). The peptides were eluted using a gradient from 100% phase A (0.1% formic acid) to 35% phase B (0.1% formic acid, 95% acetonitrile) over 43 min directly into an LTQ-Orbitrap XL mass spectrometer (Thermo Scientific). From each MS scan (Orbitrap) taken at a resolution of 60000 to detect molecules in the range of 300-1800Da, the five most abundant precursor ions were selected for fragmentation (collision-induced dissociation). The raw data files were converted to mgf files and searched with Mascot 2.2 software using Proteome Discoverer (Thermo Scientific). Peptides with a mascot probability score p<0.05 were further analysed.

### Selection of peptide for immunizations

The first six amino acids of each free end of the sequences identified by MS were regarded as a neoepitope generated by the protease in question. All protease-generated sequences were analyzed for homology and distance to other cleavage sites and then blasted for homology using the NPS@: network protein sequence analysis [50].

### Immunization procedure

Six 4–6 week old Balb/C mice were immunized subcutaneously in the abdomen with 200 μL emulsified antigen (50 μg per immunization) using Freund's incomplete adjuvant (KLH-CGG- GVAPGIGPGG (Chinese Peptide Company, Beijing, China)). Immunizations were continued until stable titer levels were obtained. The mouse with the highest titer was selected for fusion and boosted intravenously with 50 μg immunogen in 100 μL 0.9% sodium chloride solution for three days before isolation of the spleen for cell fusion. The fusion procedure has been described elsewhere [51]. The mouse work was approved by the Beijing laboratory animal administration office under approval number 200911250.

### Clone characterization

The sequence GVAPGIGPGG, named ELM-2, was selected for antibody generation. Native reactivity and peptide-binding of the generated monoclonal antibodies were evaluated by displacement of human serum in a preliminary indirect ELISA using biotinylated peptides (Biotin- GVAPGIGPGG) on a streptavidin-coated microtitre plate and the supernatant from the growing monoclonal hybridoma. Tested were the specificities of clones towards the free peptide (GVAPGIGPGG), a non-sense peptide, and the elongated peptide (GVAPGIGPGGV). Isotyping of the monoclonal antibodies was performed using the Clonotyping System-HRP kit (Southern Biotech). The selected clones were purified using Protein G columns according to manufacturer's instructions (GE Healthcare Life Science).

### Histological analysis of neoepitope distribution

Paraffin-embedded lung tissue from patients (n = 5) with fibrotic pulmonary diseases were provided by Rigshospitalet, Copenhagen University Hospital, Copenhagen, Denmark and carotid arteries (n = 5) were provided by the University Hospital, Odense, Denmark. The carotid arteries are from internal carotid endarterectomy and are intima-media tissue. The tissues were from anonymous subjects.

After paraffin melting and rehydration, the sections were pretreated with citrate buffer (citrate acid monohydrate, pH 6.0) overnight at 60° C. The sections were blocked for 30 minutes with 0.5% TBS-casein (Casein, Thimerosol, TBS). The antibodies Anti-Elastin (monoclonal BA-4, Sigma-Aldrich, diluted 1∶200), ELM mAb (2 µg/mL), ELM-2 mAb (2ug/mL), CD68 (Sigma Aldrich) diluted 1∶100, negative control Mouse IgG1 (Dako) 15ug/mL diluted in TBS were incubated overnight at 4° C. The ELM and ELM-2 monoclonal antibodies were mixed with the related free peptide 1∶10. Immunoreactivity was detected using Envision+ System-HRP (Dako). Sections were counterstained with Mayer's Haematoxylin. Pictures were taken with an Olympus DP71 digital camera on an Olympus BX60 microscope using 4 or 10× magnification.

### ELISA procedure for ELM-2

The selected monoclonal antibody was labelled with horseradish peroxidase (HRP) using the Lightning link HRP labelling kit according to the instructions of the manufacturer (Innovabioscience). A 96-well streptavidin plate was coated with 100 µL of 2.5 ng/mL Biotin- GVAPGIGPGG (Chinese Peptide Company, Beijing, China) dissolved in assay buffer (50 mM PBS, 1% BSA, 0.1% Tween-20, pH 7.4) and incubated for 30 minutes at 20°C. After washing, 20 µL of free peptide calibrator or sample were added in duplicate to appropriate wells, followed by 100 µL of 65 ng/mL conjugated monoclonal antibody, and incubated for 1 hour at 20°C. Finally, after washing, 100 µL tetramethylbenzinidine (TMB) (Kem-En-Tec) was added and the plate was incubated for 15 minutes at 20°C in the dark. All the above incubation steps included shaking at 300 rpm. After each incubation step the plate was washed five times in washing buffer (20 mM Tris, 50 mM NaCl, pH 7.2). The TMB reaction was stopped by adding 100 µL of stopping solution (1% HCl) and measured at 450 nm with 650 nm as the reference. A master calibrator prepared from the synthetic-free peptide accurately quantified by amino acid analysis was used as a standard curve and plotted using a 4-parametric mathematical fit model.

### Technical evaluation and specificity

Linearity was determined by 2-fold dilutions of quality control (QC) serum and plasma samples, as a percentage of recovery of the 100% sample. The lower limit of detection was determined from 21 zero serum samples (i.e. buffer) and calculated as the mean+3× standard deviation. The inter- and intra-assay variation was determined by 12 independent runs of 8 quality control serum samples, with each run consisting of two replicas of double determinations. The stability of serum was measured using three serum samples that had undergone freeze/thaw cycle ten times.

The ELM-2 neoepitope raised antibody was characterized using the MMP-9/12 cleaved elastin described under “*In vitro* cleavage”. The samples were diluted 1∶10 for assessment in the ELM-2 ELISA.

### Clinical relevance of ELM and ELM-2 for detection of cardiovascular diseases

The level of ELM-2 and ELM neoepitopes was assessed in serum from cardiovascular patients using the ELM-2 and ELM ELISA. The serum samples were diluted 1∶2 for the assessment. In the CVD cohort, individuals were selected and grouped on the basis of prior knowledge about the presence or absence of well-defined clinical symptoms. Importantly, pre-analytical conditions and gender- and age- distribution were similar in all groups. Hypertension was defined as patients receiving antihypertensive medical treatment and diabetes patients receiving anti-diabetic medication. Systolic and diastolic blood pressure was measured on the same day as blood sampling. Heartscore and Agatston score were calculated in the two groups of patients undergoing cardiac CT. Troponin, fibulin-1, total cholesterol, low density lipoprotein (LDL), high density lipoprotein (HDL) and triglycerides were measured prior to the blood sampling. Blood samples were drawn in tubes with EDTA and centrifuged at 200 g for 10 min. Plasma was stored at −80°C until biochemical analysis.

120 individuals with different stages of atherosclerotic heart disease were selected from two larger studies (DanRisk [52] and DEFAMI), which were actively enrolling patients at Odense University Hospital in Denmark during the same time period. The individuals were divided into four groups of 30 patients. Two of the groups were recruited from the DanRisk study; one consisted of subjects without previous cardiovascular disease (CVD) and no coronary calcium detectable by CT scan (CT-noCa); and the other group of asymptomatic subjects having no previous CVD but were diagnosed with subclinical CVD due to their severe subclinical coronary calcium shown by CT scans (CT-plusCa). From the DEFAMI study two age and gender matched groups were selected. All patients in these two groups had troponin analysis performed on suspicion of acute AMI and serial blood sampling was performed within the first 24 hours of symptom onset. Patients having non-ST elevation myocardial infarction (NSTEMI) or unstable angina were divided into a group (AMI-group), and the remaining individuals with suspected AMI, but not diagnosed, were divided into the second group (non-AMI). The diagnosis of AMI is made pursuant to the criteria for MI descibed by Thygesen el al [53] including troponin-I levels. All samples from these individuals were pre-analytically handled and stored by the analytical unit at Odense University Hospital.

The DanRisk and DEFAMI protocols were approved by the Regional Scientific Ethical Committee for Southern Denmark (S-20080140 and S-20090082) and were conducted in accordance with the Declaration of Helsinki. Written informed consent was obtained from each participant.

### Clinical relevance of ELM-2 for detection of lung diseases

The level of the ELM-2 ELISA was assessed in serum from patients diagnosed with COPD (n = 10) or IPF (n = 29) and compared with healthy controls (n = 11). The COPD and IPF serum samples were obtained as a part of the ”Lung Tissue Research Consortium” (www.ltrcpublic.com). The local IRB evaluated the study and concluded that due to the proper de-identification of samples and patients by the LTRC, an approval from the IRB was not required for this work. The controls were derived from a previously described study [54], [55] and the samples were diluted 1∶2 in the assay.

### Statistics

Comparison of serum levels of ELM and ELM-2 in the atherosclerotic heart disease cohort and the COPD/IPF cohort was performed using the two-sided non-parametric Wilcoxon rank sum test. Correlations were made by Spearman's correlation. Demographics of each patient-group were analysed by the one-way of analysis of variance (ANOVA) non-parametric Kruskal-Wallis test.

Differences were considered statistically significant if p<0.05. Area under the Receiver Operating Characteristic Curve (AUROC) was calculated using Graphpad Prism 6 software. Prism uses the method of Henley et al [56]. The standard error is calculated assuming that the area is really 0.5 as the null hypothesis and determines the p value from the normal distribution (two-tail).

## Results

### Neoepitope selection and ELM-2 ELISA characterization

The identified protease-generated elastin peptides were investigated for homology and cross-reactivity to other proteins to select sequences that were unique for elastin and the most representative of elastin degradation. The sequence GVAPGIGPGG, generated at amino acid position ‘552, was selected since it was identified by LC-MS/MS *in vitro* in MMP-9 and −12 cleaved elastin purified from human aorta, and in MMP-9 digested elastin from vascular tissue. The sequence had a very conservative C-terminal and was found in more than one peptide. The sequence was not present among the identified peptides generated by cathepsins, ADAMTSs and MMP-3 and −8. MMP-1 did generate the sequence but the fragment was only identified a single time, so MMP-9 and −12 were the predominant proteases. The sequence was named ‘ELM-2’ and selected for immunization and antibody generation.

**Figure 1:**
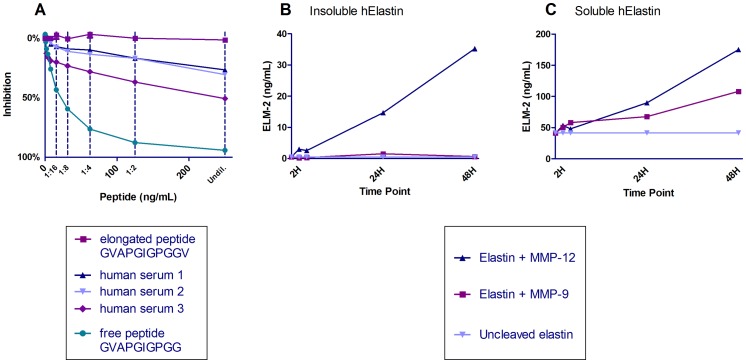
Characterization of the ELM-2 monoclonal antibody. A) ELISA showing percent inhibition of the signal of the free peptide, elongated peptide and three human serum samples. The human samples were run undiluted and diluted 1∶2, 1∶4 and so forth as indicated by the dotted lines. B+C) Release of ELM-2 by MMP-9 and −12 cleavages as a function of time of human elastin from B) insoluble elastin and C) soluble elastin. The cleaved material was diluted 1∶10 in the assay.

The requirements for selected monoclonal antibodies for ELISA development were I) IgG subtype, II) specificity towards the neoepitope and not the elongated peptide or uncleaved elastin, III) native reactivity towards diseased human body fluids and not only to the synthetic peptide and IV) acceptable dilution recoveries in human body fluids. Based on these requirements an antibody recognizing the sequence GVAPGIGPGG was selected. The subtype was determined to be an IgG2b subtype. The monoclonal antibody did not show any affinity toward either the elongated peptides or the uncleaved elastin (figure 1a). The native reactivity towards diseased human serum and plasma was high and the signal was inhibited by 50%.

By using the developed ELM-2 ELISA it was confirmed that the fragment was generated by MMP-9 and −12 (figure 1b and 1c). ELM-2 was generated when MMP-12 was added to both soluble and insoluble elastin, whereas MMP-9 was only able to generate it from soluble elastin. ELM-2 was released in a time-dependent manner. All these findings were consistent in repeated batches.

### Technical evaluation of ELM-2 ELISA

Results of the technical evaluation of the ELM-2 ELISA are presented in table 1and show a technically robust assay with dilution recovery within the recommended range of +/−20%. The accuracy and precision were acceptable with low inter- and intra-assay variation.

**Table 1:** Summary table of the technical validation of the ELM-2 ELISA.

Technical validation step	ELM-2
Target	MMP-9 and −12 degraded human elastin at amino acid number 552
Detection range/standard curve	1.82–250ng/mL
Dilution range of serum samples	1∶2 is recommended
Dilution range of plasma samples	1∶2, 1∶3 and 1∶4 is recommended
Dilution recovery of human serum*	98%
Dilution recovery of human plasma*	97%
Intra-assay variation**	6.4%
Inter-assay variation**	12%
Analyte stability***	96%
LLOQ	5.4 ng/mL

* Percentage dilution recovery was calculated as the mean of 4 human samples diluted 1∶2 and 1∶4. **Inter- and intra-assay validation was calculated as the mean variation between 8 individual determinations of each human serum sample. ***The stability of the analyte (human serum) was calculated as the mean of three different serum samples tested after freeze/thaw for one to 4 times.

### Evaluations of ELM and ELM-2 in cardiovascular diseases

**Figure 2:**
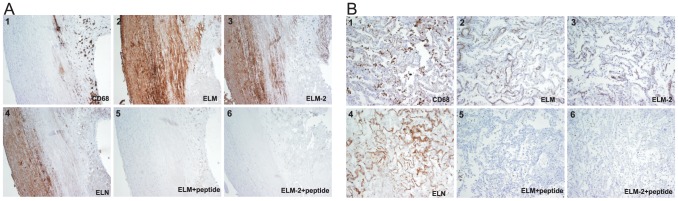
Tissue distribution of CD68, elastin (ELN), ELM and ELM-2. ELM and ELM-2 monoclonal antibodies were also mixed with the related free peptide as a control. A) Cross section of the plaque shoulder of a carotid artery from a 77 old man B) Left lung from a patient diagnosed with IPF. All pictures were taken with 10× magnification.

A representative of the histological examination of adjacent sections of diseased carotid arteries is shown in figure 2a. The elastic lamellae in tunica media were stained by elastin antibody and the plaque shoulder was stained by CD68 antibody. ELM and ELM-2 neoepitopes were localized in the disrupted tunica media and intima but not in the necrotic core. When incubated together with the epitope represented as synthetic peptide, the signal of ELM and ELM-2 was eliminated. No positive staining was found in the negative controls.

The demographics of the 120 patients in the cardiovascular cohort are shown in table 2. The only significant difference among the patient groups was the mean age, which was higher for the AMI and non-AMI groups compared to the remaining two groups.

**Table 2:** Demographical data from the cardiovascular disease cohort.

Parameter	AMI	Non-AMI	CT-plusCa	CT-noCa	ANOVA
	(n = 30)	(n = 30)	(n = 30)	(n = 30)	p-value
Age	64.5±8.5	64.2±7.9	60.3±0.3	60.3±0.4	0.00030***
Systolic blood pressure	159±28	143±26	147±20	145±14	0.030*
Diastolic blood pressure	88±15	81±14	86±11	86±10	0.19
Agatstonscore	-	-	1509±1070	-	-
Total cholesterol (mmol/L)	4.7±1.3	4.9±0.90	5.5±1.2	5.2±1.2	0.074
LDL (mmol/L)	2.9±1.2	2.7±0.8	3.3±1.0	3.1±1	0.16
HDL (mmol/L)	1.2±0.3	1.5±0.7	1.4±0.5	1.3±0.4	0.073
Triglyceride (mmol/L)	1.5±1.0	1.4±0.9	1.6±0.67	1.9±1.3	0.11
Heartscore^A^	-	-	8.3±6.2	6.3±4.5	0.061
Troponin I (µg/mL)	0.62±1.20	-	-	-	-

Values are mean ± standard deviation. The data were analysed using the one-way of analysis of variance (ANOVA) non-parametric Kruskal-Wallis test. * p<0.05; ** p<0.01; *** p<0.001,.^A^Heart score is an assessment on risk of cardiovascular disease based on age, systolic blood pressure, total cholesterol in mmol/L, and smoking status. LDL = low density lipoprotein, HDL =  high density lipoprotein, AMI = acute myocardial infarction, CT-plusCA =  subclinical coronary calcium shown on CT scans, CT-noCa =  no coronary calcium detectable on a CT scan.

**Figure 3:**
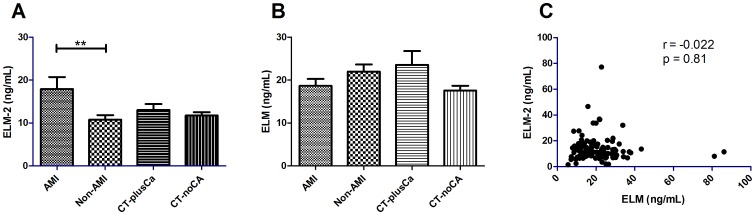
Biological validation of ELM and ELM-2 in the cardiovascular cohort. Human serum from patients with acute myocardial infarction (AMI) (n = 30), non-AMI (n = 30), coronary calcium shown on CT scans (CT-plusCA)(n = 30) and no coronary calcium detectable on a CT scan (n = 30). Bars indicate mean level. Groups were compared by Wilcoxon rank sum test. A)ELM-2, B) ELM and C) The Spearman correlation between ELM and ELM-2. Data are shown as mean±SD with 95% confidence intervals. ** p<0.01.

The serum samples from the CVD study were measured by the newly developed ELM-2 ELISA and the formerly developed ELM ELISA (figure 3) The serum levels of ELM-2 were 66% higher in patients diagnosed with AMI (mean = 18.0 ng/mL) compared to patients with suspected AMI (mean = 10.8 ng/mL) (p<0.0080). A borderline significant difference was observed between the AMI group and the group without coronary calcium (p = 0.070). Levels of ELM were not significantly different between the four groups (AMI: 18.7 ng/mL, non-AMI: 22.0 ng/mL, CT-plusCA: 23.6 ng/mL and CT-noCa:17.6 ng/mL) and no correlation was observed between ELM-2 and ELM (r = −0.022, p = 0.81).

**Figure 4:**
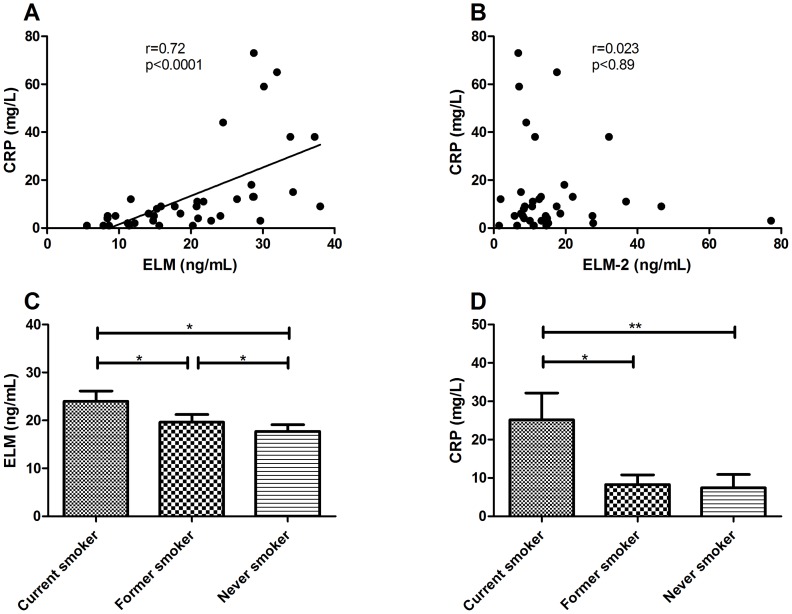
Correlations in the cardiovascular cohort. A) Spearman correlation between C-reactive protein and ELM, B) Spearman correlation between C-reactive protein and ELM-2. * p<0.05, C) Biological validations of ELM versus smoking habits, D) Biological validations of C-reactive protein versus smoking habits. * p<0.05; ** p<0.01; C-reactive protein was only measured in the acute myocardial infarction (AMI) and non-AMI groups.

While a small correlation was found between ELM and HeartScore (r = 0.19, p = 0.045), the ELM and ELM-2 showed no significant correlations with age, systolic and diastolic blood pressure, Agatston score, total cholesterol, LDL, HDL, triglyceride and fibulin-1 (data not shown). The levels of the two elastin degradation markers neither showed significant correlation with troponin I levels nor did they correlate with the risk factors of hypertension, diabetes and hypercholesteremia (table 3). However, smoking habits had an impact on the ELM levels (figure 4c). The mean level of ELM in current smokers (24.0 ng/mL) was significantly elevated compared with those of former smokers (19.7 ng/mL, p = 0.025) or subjects who had never smoked (17.7 ng/mL, p = 0.013). The level of ELM in former smokers was also significantly higher than that in never smokers (p = 0.048). Interestingly, a significant mean elevation of ELM-2 was observed in former smokers (15.7 ng/mL) compared with current smokers (10.4 ng/mL). The mean levels of C-reactive protein (CRP) measured in the AMI and non-AMI groups showed a highly significant correlation with ELM (r = 0.72, p<0.0001) (figure 4a) but not with ELM-2 (r = 0.023, p = 0.89) (figure 4b). CRP was higher in current smokers than in never smokers (p = 0.0063) and former smokers (p = 0.013) (figure 4d).

**Table 3:** Differences in elastin neoepitopes levels compared to patient parameters presented with p-values. n>5.

	Parameter		ELM	ELM-2
			All patients	All patients
**Risk factor**	Sex		0.65	0.31
	Hypertension		0.47	0.94
	Diabetes		0.22	0.94
	Hypercholesteremia^A^		0.070	0.88
	Smoking^#^	- Current/Never	0.013*	0.34
		- Former/Never	0.048*	0.15
		- Current/Former	0.025*	0.0064**
**Treatment**	Statins		0.29	0.66
	Angiotensin-converting-enzyme inhibitor		0.63	0.42
	Angiotensin II receptor antagonist		0.43	0.28
	Beta-blocker		0.17	0.57
	Calcium channel blocker^B^		0.057	0.71
	Thiazide^C^		0.070	0.13
	Loop-diuretics		0.52	0.13

* p<0.05; ** p<0.01; ^A^Mean of ELM release is 17,2 ng/mL for patients having hypercholesteremia and 21,3 ng/mL for patients not having it. ^B^Mean of ELM release is 25.8 ng/mL for patients taking Calcium channel blocker and 19,4 ng/mL for patients not taking the drug. ^C^Mean of ELM release is 19,6 ng/mL for patients taking thiazides and 20,6 ng/mL for patients not taking the drug. ^#^Levels of mean of ELM versus smoking habit is shown in Figure 4.

No correlation was observed between the type of cardiovascular medications and ELM or ELM-2 (table 3). A borderline significant correlation was however observed between ELM and calcium channel blockers (p = 0.057), where the mean level of ELM was 25.8 ng/mL in patients taking the treatment compared with 19.4 ng/mL for patients not receiving calcium channel blockers.

To investigate the diagnostic value of ELM, ELM-2 and previously demonstrated cardiac markers, ROC values were calculated (table 4). ELM-2 had the best diagnostic value in AMI patients compared to non-AMI (AUC = 70%, p<0.0078). Other markers showing a separation of the two patient groups were HDL (AUC = 69%, p<0.020) and systolic blood pressure (AUC = 67%, p<0.022). The diagnostic value of ELM was fairly low (AUC = 61%, p<0.15).

**Table 4:** Diagnostic power of each biomarker for the separation of patients with AMI compared non-AMI.

Parameter	AMI vs non-AMI		
	AUROC	Std.error	p
Troponin	0.92	0.039	<0.0001***
ELM-2	0.70	0.069	0.0078**
HDL	0.69	0.074	0.020*
Systolic blood pressure	0.67	0.071	0.022*
Diastolic blood pressure	0.63	0.073	0.088
ELM	0.61	0.074	0.15
Osteoprotegerin	0.61	0.075	0.16
C-reactive protein	0.59	0.10	0.36
Heartscore	0.58	0.080	0.29
Triglyceride	0.57	0.081	0.40
LDL	0.56	0.080	0.45
Ostepontin	0.55	0.076	0.50
Total cholesterol	0.52	0.079	0.79

AUROC =  Area Under the Receiver Operating Characteristic Curve. Data are show as the AUROC, a probability of correct diagnosis by each marker. The P value indicates significance of the AUROC diagnosis compared to the null hypothesis which is an area of 0.5.

* p<0.05, ** p<0.01, *** p<0.01.

### Evaluations of ELM and ELM-2 in pulmonary diseases

A representative of the histological examination of adjacent sections of fibrotic lung tissue is seen in figure 2b. It showed that elastin, ELM and ELM-2 were localized in thin fibres throughout the respiratory tree and in blood vessel walls. When incubated together with the neoepitope represented as a synthetic peptide, the signal was eliminated. CD68 staining of macropahges was observed in the lumen of alveolus. No positive staining was found in the negative controls.

**Figure 5:**
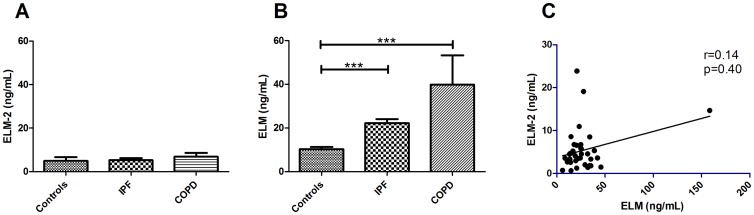
Biological validation of ELM and ELM-2 in pulmonary diseases. Human serum from patients with chronic obstructive pulmonary disease (COPD)(n = 10) and idiopathic pulmonary fibrosis (IPF)(n = 29) compared with controls (n = 11). Bars indicate mean level. A) ELM-2, B) ELM (Data have been published with permission from Skjøt-Arkil et al [45], C) The Spearman correlation between ELM and ELM-2. Groups were compared by Wilcoxon rank sum test. Data are shown as mean±1.8SD with 95% confidence intervals. *** p<0.001.

Mean levels of ELM-2 were not elevated in patients with COPD (6.9 ng/mL) or IPF (5.3 ng/mL) compared to controls (4.9 ng/mL) (figure 5). Levels of the two neoepitopes in lung tissue were not correlated (r = −0.14, p = 0.40).

To investigate the diagnostic potential of ELM and ELM-2 in the lung disease cohort, ROC values were calculated (table 5). The diagnostic value of ELM-2 (AUC = 69%, p = 0.15 and AUC = 59%, p = 0.40) was lower compared to that of ELM (AUC = 97%, p = 0.00025 and AUC = 90%, p = 0.00011) in COPD and IPF patients, respectively.

**Table 5:** ROC values of ELM and ELM-2 in the lung disease cohort.

Para meter	Control vs COPD			Control vs IPF		
	AUROC	Std.error	p	AUC	Std. error	p
ELM^A^	0.97	0.032	0.00025***	0.90	0.048	0.000 11***
ELM-2	0.69	0.13	0.15	0.59	0.12	0.40

*** p<0.01, ^A^Data modified from Skjøt-Arkil et al [45], COPD = chronic obstructive pulmonary disease, IPF = idiopathic pulmonary fibrosis.

## Discussion

This study shows that two different MMP generated fragments of elastin were differently related for two different diseases. Our data indicated that in cardiovascular tissue, elastin degradation by MMP-9 and-12 leads to the release of the ELM-2 neoepitope while the degradation in pulmonary diseases leads to the release of the ELM neoepitopes.

Elastin degradation has been investigated by several groups [57]–[59]. Even though elastin is one of the most stable proteins in the body and has a half-life of several decades, some age-related turnover of mature elastin occurs in normal adults as indicated by low but detectable levels of desmosine [24], [60]. The neopitope ELM-2 identified in this study was predominantly generated by MMP-12, whereas MMP-9 was only able to degrade soluble elastin. This is an important characteristic of the ELM-2 fragment, as it implies that detection of MMP-9-generated ELM-2 can only be made if elastin previously has been degraded into the soluble form. MMP-9 activity is therefore dependent of the solubility of elastin. However, as observed by others, the cleavage products are dependent on incubation time, amount of protease and the stability of the peptide [57]. The solubilised elastin used for the *in vitro* cleavage are not identical to the *in vivo* found tropoelastin since it is generated by oxalic acid and therefore still crosslinked with desmosines. Taddese et al. and Heinz et al. both describe MMP-12 generated cleavage sites at amino acid ‘441 and ‘552 in tropoelastin thus we believe that ELM and ELM-2 may also derive from tropoelastin [61], [62]. The same degradation profile as the one for ELM-2 has been shown for ELM [45]. In a similar fashion, it has been described for type I collagen, where the collagenases MMP-1 and −13 or cathepsins K are needed to degrade native type I collagen before other proteases such as the gelatinases MMP-2 and −9 may further degrade the protein [63]–[67].

The histological analysis of the neoepitopes distribution revealed that ELM and ELM-2 are located in the same areas as the total elastin and that macrophages are mainly present in the plaque shoulder in arteries close to the ELM and ELM-2-stained fragmented elastic lamellae. The same co-localization was observed in the diseased lung tissue, where macrophages were located in the alveolus air spaces close to the fibres. Since macrophages are the main source of MMP-12 [38], it emphasizes that ELM-2 and ELM are generated by MMP-12 at the site of injury in both tissues. Theoretically ELM and ELM-2 should be a proper subset of the total elastin, however, the applied commercial monoclonal elastin antibody was described by the manufacturer to bind insoluble elastin and tropoelastin but also to bind proteolytically generated elastin fragments. Thus elastin, ELM and ELM-2 antibodies all bind to elastin fragments. The distribution of the staining of ELM, ELM-2 and elastin, however, was different, suggesting they provide different information. Quantification by ELISA was able to distinguish the levels of ELM and ELM-2 in serum from patients with cardiovascular and pulmonary diseases, which the histological analysis were not. The reason, that the difference between the location of the two neoepitopes in immunohistochemical staining was not more clearer is that both neoepitopes are generated in lung and arterial tissue and are detected by immunohistochemical stainings, but the release of the neoepitopes into the circulation is different in the two tissues; ELM-2 is mainly released from arteries whereas ELM is mainly released from lung tissue. This difference was quantifiable by ELISA. Another difference between the two methods is, that in serum the proteins are in their native form while in the paraffin-incubated tissue the proteins are denatured making the antibody bind to a greater proportion.

The cardiovascular samples relied on a well-characterized cohort in which all samples were collected under the same standard procedure and covered patients with various degrees of atherosclerosis, from seemingly none to chronically ill. The non-AMI group however, comprised of heterogenous patients whom had been suspected to have had an AMI, when first arrived at the hospital. It is not known if they instead had atherosclerosis, renal failure, myosis or sepsis. Some of the non-AMI patients had different degrees of CVD and a fraction had heart failure, all of which might have influenced their biomarker levels. The patients without any coronary calcium are the least diseased group and functioned as controls in our analysis. A borderline significant difference between the AMI group and the no calcium group suggests that a study in a larger patient group and thereby with more statistical power could confirm this difference. The level of ELM-2 was not elevated in the CT-plusCA group, which would have been expected for an atherosclerotic marker. This questions if ELM-2 might be a marker of acute heart damage. The specific activity of MMP-9 and −12 and their effect on cardiac remodeling during either physiology or pathology have to date not been described in the literature. MMP-12 expression in cardiac valves taken from patients suffering from infective endocarditis has been shown to be elevated [68] and human and animal studies have established the presence and activity of MMP-9 on cardiac-related remodeling [69]–[72].

One thing that might differ in the four CVD groups is drug intake, which could influence the levels of a biochemical marker. The patients included in the cohort received various cardiovascular medications such as statins, beta blockers, diuretics and antihypertensives. However, none of these treatments correlated significantly with ELM or ELM-2. Calcium channels blockers, though, did show a borderline significant difference with ELM levels, but not with ELM-2, which could be explained by a different effect of the blockers on pulmonary arteries.

ELM and ELM-2 were not found to correlate with the cardiovascular risk factors which imply that ELM and ELM-2 are independent of these factors. The lack of correlation may also be attributed to the fact that the ELM and ELM-2 assays measure MMP-degraded fragments of elastin and not the total protein as do many of the other CVD biomarkers. This protein-protease combination may make the neoepitopes more disease-specific and reveals different profiles of the remodelling in cardiovascular and pulmonary diseases. This has been shown in other diseases such as in patients with ankylosing spondylitis, where the degradation markers of CRP, the CRP-MMP and CRP-Cat, had a higher diagnostic value than the total CRP [33]. It is also well appreciated in cartilage pathologies that aggrecanases and MMP-mediated cartilage degradation provide different molecular information [73]. While the amount of intact protein may not change in a particular pathology, the protein fingerprint may differ dramatically [29]. This disease specificity is believed to be the reason for the high diagnostic value of ELM-2 compared to the other CVD markers. Troponin, a common clinical AMI marker [74], was used to divide the patients in the DEFAMI-study, making it the reference for the other markers and the one with the highest ROC values. Since troponin is very site specific and no correlation to ELM-2 was observed, it questions the possibility of ELM-2 as a marker of acute heart damage. It is possible that AMI is a disease that is not as localized as one might believe, and therefore involves other vessels than the cardiac. ELM-2 reflects the overall damage and not just heart damage. By the histological analysis it was also shown, that ELM-2 is not restricted to cardiac arteries since it was localized in carotid arteries. This could explain why ELM-2 is a marker of AMI but not correlates to troponin. The HeartScore, which combines smoking, age, gender, blood pressure and lipids, is widely used for risk estimation. However the score had a low diagnostic value and HDL and blood pressure even had a better value when not combined into the score. Osteopontin has been reported to be a prognostic biomarker of heart failure [75], [76] but it is expressed in many tissues and therefore has been described as a marker of other pathologies [77]–[79]. This multiple expression might be the reason for its low diagnostic value in this study. Even though osteoprotegerin has been described to have a significant role in the pathophysiology of atherosclerosis and has been reported elevated in patients with AMI [80], [81], its diagnostic potential in this study was found to be not among the highest.

The diagnostic power of ELM and ELM-2 in the COPD/IPF cohort reinforces the different profiles of the two elastin fingerprints. In patients with pulmonary diseases, ELM levels were up to 575% higher than the levels of ELM-2. One major limitation of the current COPD/IPF study was the relatively small sample size and the limited clinical information obtained. Thus, these preliminary findings need to be validated in larger clinical settings for the diagnostic utility.

One complicating factor in the use of ELM and ELM-2 is that elastin is expressed in the arteries, lung tissues, skin and tendons [1]. Thus, several co-morbidities may influence the systemic levels of ELM and ELM-2, as seen with smokers in the cardiovascular study. Smoking affects CRP and ELM and might be the reason for the low diagnostic potential of these two markers in AMI patients. People with COPD are also at increased risk of acute cardiovascular events [82], which will influence their serum levels of ELM and ELM-2 and thus their ratio. Further investigations are needed to determine each disease's contribution to the total pool of the two elastin neoepitopes.

The tissue-specific alternative splicing of tropoelastin leads to variations in incorporation of exons or coding regions into mRNA and therefore in the production of elastin isoforms. This might be one of the reasons for cleavage at amino acid position ‘441 in pulmonary diseases generating ELM and cleavage at amino acid position 552 in cardiovascular diseases. The neoepitope technology, measuring specific protein degradation fragments, allows for assessment of specific proteolytic activity in given tissues, provided that the sequence is unique for one or a few proteases. Ultimately, a panel of biomarkers may be needed to characterize different aspects of cardiovascular and pulmonary diseases in patients, including prognosis, diagnosis and assessment of the efficacy of intervention.

In conclusion, the newly identified ELM-2 was highly related to cardiovascular diseases and might be a potential non-invasive biomarker of AMI. ELM is, in contrast, related to lung tissue degradation, indicating that elastin degradation by MMPs at different amino acid sites may provide different biological information and degradation profiles most likely depending of the disease in question. Further testing in a larger cohort is needed to confirm these preliminary findings.
